# Benefits and payments for research participants: Experiences and views from a research centre on the Kenyan coast

**DOI:** 10.1186/1472-6939-13-13

**Published:** 2012-06-22

**Authors:** Sassy Molyneux, Stephen Mulupi, Lairumbi Mbaabu, Vicki Marsh

**Affiliations:** 1 Health Systems and Social Science Research Group, Kenya Medical Research Institute/Wellcome Trust Research Programme, P.O. Box 230, Kilifi, Kenya; 2 Centre for Tropical Medicine, University of Oxford, OX3 7LJ, Oxford, UK; 3 The Ethox Centre, Department of Public Health and Primary Health Care, Oxford University, OX3 7LJ, Oxford, UK; 4 Child and Newborn Health Group, Kenya Medical Research Institute/Wellcome Trust Research Programme, P.O. Box 43640 – 00100, Nairobi, Kenya; 5 Kenya Medical Research Institute/Wellcome Trust Research Programme, P.O. Box 230, Kilifi, Kenya

## Abstract

**Background:**

There is general consensus internationally that unfair distribution of the benefits of research is exploitative and should be avoided or reduced. However, what constitutes fair benefits, and the exact nature of the benefits and their mode of provision can be strongly contested. Empirical studies have the potential to contribute viewpoints and experiences to debates and guidelines, but few have been conducted. We conducted a study to support the development of guidelines on benefits and payments for studies conducted by the KEMRI-Wellcome Trust programme in Kilifi, Kenya.

**Methods:**

Following an initial broad based survey of cash, health services and other items being offered during research by all programme studies (n = 38 studies), interviews were held with research managers (n = 9), and with research staff involved in 8 purposively selected case studies (n = 30 interviewees). Interviews explored how these ‘benefits’ were selected and communicated, experiences with their administration, and recommendations for future guidelines. Data fed into a consultative workshop attended by 48 research staff and health managers, which was facilitated by an external ethicist.

**Findings:**

The most commonly provided benefits were medical care (for example free care, and strengthened quality of care), and lunch or snacks. Most cash given to participants was reimbursement of transport costs (for example to meet appointments or facilitate use of services when unexpectedly sick), but these payments were often described by research participants as benefits. Challenges included: tensions within households and communities resulting from lack of clarity and agreement on who is eligible for benefits; suspicion regarding motivation for their provision; and confusion caused by differences between studies in types and levels of benefits.

**Conclusions:**

Research staff differed in their views on how benefits should be approached. Echoing elements of international benefit sharing and ancillary care debates, some research staff saw research as based on goodwill and partnership, and aimed to avoid costs to participants and a commercial relationship; while others sought to maximise participant benefits given the relative wealth of the institution and the multiple community needs. An emerging middle position was to strengthen collateral or indirect medical benefits to communities through collaborations with the Ministry of Health to support sustainability.

## Background

Debates on the ethics of international health and health research have shifted over the last twenty five years away from a focus on the relevance and value of informed consent, towards considering broader challenges such as the potential for exploitation in international research, the need to make research responsive to local needs of host communities, and the implications of research for international relations and law [1,2]. This shift is related to growing recognition that focusing on the protection of research participants through reviewing research proposals before they begin is inadequate; that there is a need to look beyond the design of studies and to see how research is actually being conducted on the ground [2]. The importance of social science studies for understanding the dilemmas that are faced and generated, and the ethical implications of how these are resolved, have begun to be highlighted [3]. Depending on their design, such studies can fall within the spectrum of approaches termed ‘empirical ethics’ [4,5], and may incorporate deliberative elements [6].

Within this general movement in ethical focus, there is consensus that unfair distribution of the benefits of research is exploitative and that - as a moral wrong - it should be minimised. One approach to doing this is to provide ‘fair benefits’ to participants and their communities [7,8]. There is still debate over what constitutes fair benefits, and over the appropriate balance in benefits between micro level issues of justice and broader social determinants of health at the macro level [7-13]. However, increasing benefits to participants and communities involved in research is widely agreed as one approach to minimize exploitation.

The fair benefits framework distinguishes between benefits from both *the conduct* and *results* of research, and between:

· *direct benefits* to those enrolled in the research (for example diagnostic tests, distribution of medications and evaluation services); and

· *collateral or indirect benefits* not targeted specifically at those involved in the research (for example providing antibiotics for respiratory infections, health service provision, digging of bore holes for clean water, or research capacity building) [7,8,14]. Beneficiaries of collateral benefits might be research participants, other identifiable individuals such as family members, or the general community.

In considering fair benefits for research, a widely accepted ethical condition is that the research must pose few risks to individual participants, or the benefits to them should outweigh the risks [7]. Where potential risks outweigh benefits to participants, the social value of the research should justify the risks [15,16]. It is also increasingly argued that the risk-benefit ratio for the communities within which the research is conducted should be favourable [15]. However, the exact nature of the benefits that can and should be provided for various studies in different settings, and their mode of provision, remain ill-defined and often strongly debated. The boundary between ‘benefits’ and obligations, for example with regards to ancillary-care in health, is also complex and contested [17-19], as will be returned to in the discussion of this paper.

The notion of undue inducement, and the paradoxical relationship with exploitation, has received particular attention in benefits debates. As Koen et al. [20] have argued, inducement by itself can be ethically justifiable, even if it contributes to participants doing something that they might otherwise not have done. However inducement becomes ‘undue’ where an excessive offer distorts decision-making, leading to individuals participating against their better judgment. Also of concern with regards to inducement is the potential to disproportionately attract the poor, and the fabrication of information in order to access study benefits. The dilemma, raised by Macklin (1989) and summarized by Ballantyne is: ‘*offer participants too little and they are exploited, offer them too much and their participation may be unduly induced*’ [21]; p 179. This paradox is particularly stark in international collaborative research, where research institutions and bodies may be relatively wealthy, operating in and among relatively low income settings and populations.

One specific form of benefit in research is payment of cash. Cash payments which reimburse or compensate for time and inconvenience are not considered benefits, and are widely accepted in international guidelines. For these forms of payment, the challenge is the limited amount of operational guidance to set appropriate payment levels. More controversial are cash payments as *incentives* to participate in research, or as *appreciations* for contributions to the research [20]. The specific additional concern about money as a benefit (beyond undue inducement) is the potential to commercialize an altruistic endeavor. Those in favour of financial payments argue that altruistic motives are not incompatible with receiving pay, that research has long been commercialized for other research stakeholders, and that participants are not only motivated by money [20]. A particular concern expressed informally in low income settings is that populations should not be deprived of potential payments simply because of their poverty as this leads to a double inequity: both poverty and inability to benefit financially.

Debates on benefits and payments in all low income settings are hampered by relatively little information on what is currently happening on the ground, and on the range of stakeholders’ views on current practice. An exception is work by Lairumbi et al. [22-24] which explores perceptions of, and experiences with, benefits among a diverse range of research stakeholders across Kenya. In this paper we present a separate but complementary set of data. We explore views and experiences with regards to benefits and payments from a diverse range of studies conducted by one large and long-term multi-disciplinary research programme in Kenya - the Kenya Medical Research Institute (KEMRI)-Wellcome Trust Research Programme in Kilifi - and consider the implications for institutional guidelines aimed at ensuring fair involvement of participants. We focus on the direct and collateral ‘benefits’ (all cash, health services and other items) offered to study participants and to community members over the course of the conduct of studies, as opposed to post study benefits, or aspirational benefits.

## Methods

### KEMRI- Wellcome Trust Programme in Kilifi

The KEMRI-Wellcome Trust Programme is a collaboration between the KEMRI Centre for Geographical Medicine Research, Coast (CGMRC), and the Wellcome Trust, UK. KEMRI is a parastatal organisation under the Ministry of Health (MoH), with 10 research centres in Kenya, of which the CGMRC is one of the largest. The KEMRI-Wellcome Trust research programme (KEMRI/WT) was established in 1989. It has grown enormously over the last twenty years, now employing over 800 staff between sites in Nairobi and Kilifi, and conducting a wide range of interdisciplinary research including clinical, basic science, epidemiological and public heath aspects of major childhood and adult diseases of concern in Kenya. A core aim of the programme is strengthening regional capacity to conduct and lead internationally competitive research.

This paper focuses on work conducted in the largest of the two sites, Kilifi. Kilifi district is on the coast of Kenya, with residents primarily from the Mijikenda ethnic group. There are very high levels of poverty, and low levels of literacy across the district. As described in greater detail elsewhere [25], a key feature of the Kilifi work has been its’ deliberate development within a District Hospital, with much research being carried out in a “real world environment” serving a rural community. The research centre provides support to the hospital to ensure a good standard of care is available to those using the departments where research is conducted, regardless of their involvement in research. This is a form of collateral benefit to the general community as a result of the research programme having a long-term presence in the area, with the additional resources including medical and clinical officers, paediatric drugs and equipment and a paediatric intensive care ward. Within the community, clinical services are supported at specific government health centres and dispensaries.

Every study carried out by the programme is scrutinised in advance by local and independent national and international scientific and ethical review committees. Over the last ten years, informed by social science research activities (see for example [25-29]), both programme wide and study specific community engagement activities have been strengthened. For example a large network of KEMRI community representatives elected by community members has been established, and the amount and type of interactions between research staff and community leaders and ‘ordinary’ community members has been increased. Interactions have increased in communities and within the research programme and hospital.

The research presented in this paper was initiated out of an institutional interest in developing locally appropriate guidance on benefits and payments for the diverse range of studies conducted in Kilifi. From the outset, it was clear that relatively high risk studies – such as phase 1 and 2a trials - needed separate consideration with particularly careful scrutiny on benefits and payments by the Ethics Review Committee. These studies were therefore not discussed and are not included in the considerations outlined in the rest of this paper.

## Methods

Data collection was in two main phases: an initial audit of current practice and experiences and views from front-line staff involved in distributing benefits; and a workshop involving a wide range of research staff and some MoH managers.

### Audit of current practice

The audit involved an initial email survey followed by semi-structured interviews with principal investigators and interface staff involved in eight purposively selected case studies. These data were supplemented by interviews with key informants expected to have relevant cross-study information.

#### Email survey

56 principal investigators leading active Kilifi based studies were contacted to identify studies that were still in recruitment, data collection, analysis or write-up stages. Semi-structured questionnaires were filled for each of the 38 active (sub) studies identified (n = 24 PIs). The questionnaire for these studies covered: the nature of services, payments and other items (‘benefits’) offered to participants, the intended recipients of benefits, stage of study at which benefits are given, and any community benefits.

#### In-depth interviews

On the basis of the data gathered above, eight studies were purposefully selected as case studies. Selection was based on maximising diversity among the case studies with regards to: duration of participant involvement; age and health status of study participants; whether studies were facility or community based; whether studies were based only in Coast Province or multi-centre international studies; level of risk in studies; and whether any money was given to participants. A summary of the case studies is presented in Table 1. Study PIs and/or their Project managers (n = 11) were individually interviewed while the fieldworkers of two studies (n = 19) participated in two FGDs. Interviews explored in more detail what benefits were given to whom and why, how benefits are communicated to participants and wider communities, and their experiences, views and recommendations with regards to benefits and payments.

**Table 1:** Case study details

**Study**	**Overall duration**	**Start date**	**Study participant duration**	**Targeted participants**	**Description of study**	**Study site**
**Perfusion study (PS)**	2 years	July 2008	2-6 months	400 children	· A RCT, severely ill, admitted with shock. Venous blood draws: admission, 8 hrs, 24 hrs& 48 hrs	**Hospital**
					· Follow up (f/up) day 28/60 after discharge	
**TB study**	2 years	Sep 2009	6 months	1500 children	· Severely ill, inpatient children with suspected TB. All children managed using WHO standards.	**Hospital**
					· Venous blood samples taken; 1 or 2 f/ups decided upon by clinician	
**Respiratory infections I (RSV I)**	4 months	Jan 2009	Approx 20-30mins	400 children	· Study to evaluate nasopharyngeal sampling tool	**Community Health Centre**
					· Nasal samples collected using swabs/nasal wash	
**Malaria Vaccine (MV)**	3 years	Jan 2009	3 years	1000 children	· A large Multicenter Vaccine trial. Trial vaccines injected 3 different times; 5 venous blood draws.	**Community**
					· F/ups at homes every 6 days after injections; monthly follow up between injections	
**Immunology (Immuno)**	5 years	2006	5 years	5000 children	· Cohort study of natural malaria immunity in children, recruited at birth	**Community**
					· 1 annual blood draw but a 5 ml sample at any fever event	
					Weekly f/up visits at homes by FWs.	
**Respiratory Infections II (RSV II)**	4 months	Oct 2009	4 months	50 household members	· A study to identify pattern of infection	**Community**
					· Children who were born in previous epidemic and have elder sibling	
					· Nasal swabbing twice a week at participants’ homes; saliva samples once a week	
**HIV study (HIV)**	10 years	2006	>2 years	30	· Men who have sex with men (MSM), enrolled in a longitudinal observational HIV study.	**Community**
					· F/ups: weekly-month 1, Bi-weekly-month 2, then monthly for minimum 2 years	
					· 50 ml blood drawn per f/up	
**Verbal autopsy (VA)**	>2 years	July 2008	30-45 mins	1000 p.a.	· Relatives of deceased persons interviewed (30-45mins)	**Community**
					·Interviews done at participants’ homes	

Key informant interviews (KIs)

Individual and group interviews covering similar topics were held with nine key staff members including community facilitators, the head of clinical trials, and the research coordinator based at a dispensary in the district where there has been significant research activity.

### Consultative workshop

On the 15^th^ of December 2009, a workshop to discuss current and future policies and practices around payments and benefits to research participants and communities for KEMRI/WT studies was held in Kilifi. 48 people attended the workshop, representing different cadres of staff at the research centre, including senior and mid-career scientists, research officers, community liaison staff, field workers, doctors and nurses. Non-KEMRI participants included the District Medical Officer (DMO) and nursing officers representing Kilifi District Hospital. Professor Mike Michael Parker, a bioethicist and Director of the Ethox Centre at Oxford University, UK, facilitated the planning of the workshop and the final plenary discussion.

Given that an important objective of the workshop was to propose approaches to payments and benefits for different types of research in Kilifi, the workshop included : 1) presentation of a review of relevant literature and the findings of the above audit; 2) six small group discussions bringing together staff with similar experience on appropriate individual and community benefits for a series of hypothetical studies designed on the basis of the audit (Table 2); and 3) a final plenary session where issues raised in the small groups were discussed in more detail. For the small group discussions, topics allocated to specific groups to discuss in more detail in relation to each scenario outlined in Table 2 were:

· Compensation for travel: Groups 1 and 2: field staff and community facilitators

· Medical benefits to participants: Groups 3 and 4: research assistants and medical staff

· Collateral benefits to communities: Groups 5 and 6: senior researchers and MoH managers

**Table 2:** Tasks and hypothetical cases for groups

**Task 1: When is compensation due? (All groups)**	**Task 2: Scenarios (one per group)**
‘It is often said that research should try to balance benefits and costs/risks to participants such that they are not made worse off by participation, including that they do not spend their own money, use their own time or experience inconvenience that they would not otherwise have. Do you agree with this? Why or why not? If you agree, how much cost or inconvenience requires some kind of compensation? For each of the four research situations below, discuss the following issues:	**1:** In depth interviews about health beliefs taking at least 2 hours
	**2:** Interview taking 30–45 mins with mother and finger prick blood sample from a well infant on one occasion only
	**3:** Vaccine study. In addition to screening and vaccination visits, intervention and control groups are asked to attend for follow up visits 6 times over 2 months following vaccination then 6 monthly for 2 years. Free medical care for acute intercurrent illnesses is provided for study children 2 years.
	**4:** Clinical trial. A study on KEMRI ward of a particular type of treatment for severely ill children. After discharge, the child should be brought back for follow up once to KEMRI OPD where a single small venous blood sample will be taken and any health issues the child has will be addressed.
-Is any compensation due? Why or why not? What would make a difference to your views on whether compensation is due?	
-If yes, broadly what type of compensation should this be?	
	**5:** Field study with well children. A study where a group 200 children under 5 years in the same area will be followed up for 5 years to assess development of immunity to malaria. Children are visited weekly to check for fevers, medical care is provided free at the nearest health dispensary for all acute intercurrent illnesses for study children and once a year a venous sample is taken. If children become unwell during the study and cannot be managed at the dispensary, they will be asked to travel to KDH for further treatment.

In each case, the specific areas of interest for discussion about each type of study were: should this benefit be given, and if so what specifically should be given, how and to whom? Groups were also asked to consider whether there should be any flexibility and how would this be managed.

Following the workshop, a draft report was circulated to all participants for their comments and inputs, and issues documented as needing further resolution taken up by a small group consisting of two experienced community facilitators in the research programme (who are local residents, including the community liaison manager), a clinical trials manager (who is also a clinician), and two social scientists (SM and VM).

### Data management and analysis

All audit interviews were audio-recorded and transcribed verbatim shortly thereafter. Three authors independently identified key themes, and following discussion agreed on themes to group key data. Summaries of data were used to develop the ideas and discussion guides for the workshop. At the workshop, notes were made by specially allocated note takers from both the small group and plenary discussions. These notes were drawn upon by the same three researchers to highlight areas of agreement, and areas of debate and disagreement requiring further discussion or research by the small group formed to take forwards discussions and agreements post workshop.

### Findings

In this section we bring together the data from the audit and the workshop, to illustrate what is happening on the ground in Kilifi regarding each major form of benefit or payment, including challenges and negative impacts, and views on what should happen. Following an overview of types and levels of benefits across all studies, we look in turn at experiences with: medical benefits, travel costs, other benefits and compensation for time. The cross-cutting issues relevant to developing guidelines, including the importance of community level collateral benefits in low income settings, and the need for flexibility and careful consideration of communication issues in policies and procedures, are highlighted in the discussion.

### Types and levels of benefits offered

From reported data in the audit, with the exception of the verbal autopsy study, which involved a 30–45 minute interview only with adults in their homes, all studies offered some form of benefit or payment to participants. Table 3 shows the benefits and payments given by the seven studies, illustrating not only the diverse range, but also the centrality of direct and collateral medical benefits, and the reimbursement of travel expenses. Other types of benefits included provision of food for individuals, and other small gifts such as books and pens. In general, the levels of collateral benefits for individuals increased with studies seeking to involve participants for longer, and where an (experimental) intervention was involved. For example the benefits for individuals in the malaria vaccine study (MV) and the Immunology study (Immuno) were greater than that of the TB study or the RSV study.

**Table 3:** Benefits provided by specific studies (beyond standard of care locally)

**Study**	**Direct medical benefits for study participants**	**Collateral medical benefits**	**Transport/fares**	**Other benefits**
**Perfusion study (PS)**	· Hourly monitoring of patient, potentially leading to more prompt identification and treatment of other acute illnesses	· Additional emergency and triage training (ETAT) for all study staff on high dependency unit (HDU)	· Fare for follow-up on days 28 and 60 after discharge	· 100$ shillings for lunch on days 28 and 60 day follow-up visits
		· Lab and clinical equipment support to health facilities		
	· Participants do not queue at hospital when sick between follow-ups			
	· Potentially more rapid referral to provincial referral hospital			
**TB study**	· Free management of TB, contact-tracing and management of infected adults	· Enhancing diagnostic and laboratory capacity of clinic	· Fare for follow up as determined by clinicians	· None
**Malaria Vaccine (MV)**	· Free treatment of all acute infections including payment of hospital bills	· Physical upgrading of dispensaries in which trial is taking place, and laboratory clinical support including provision of vaccines where necessary	· Fares or lifts in KEMRI cars to hospital for medical care	· Refurbishment of facilities
	· Regular screening of children for anaemia, de-worming			
			· KEMRI cars also help in times of emergencies	
	· Referral of chronic illnesses and those that cannot be handled at the facility			
		· Resuscitation equipment for use by all dispensary patients		
	· Uninterrupted access to EPI & rabies vaccines, and Hep B even in cases of MOH stock-outs			
		· Study personnel man facility when MOH staff are away		
**Immunology (Immuno)**	· Weekly testing of children for fevers	· Study personnel man facility when MOH staff are away	· KEMRI cars or taxis sent to take sick participants to hospital (day/night respectively)	· Milk, bread for children during annual bleeds.
	· Prompt, timely treatment of all acute illnesses			
				· Notebooks and pens for the children
		· Disease surveillance for the Ministry of Health		
	· Referral of chronic cases to government facilities			
**RSV 1**	· Free treatment of all acute infections	· Lab and clinical equipment support to health facilities	· N/A (participants came to facility for own reasons)	· Sweets for children
		· Antibiotics and gloves supply for community facilities		
		· Disease surveillance for the Ministry of Health		
**RSV 2**	· Free examinations, medical treatment of all acute infections		· Fares to hospital for treatment	· None
	· All medical bills settled by the study			
**HIV**	· Free screening & treatment for STI’s, HIV tests even in the absence of symptoms	· Access to services without stigma as a result of careful training of all staff	· 600 ($1.30) shillings fares provided at a flat rate	· Free lubricants
				· Food tickets for those on ARVs and the very poor
	· ART started at earliest possible time			
	· HIV disease monitoring and support counselling			
	· Free condoms and Hep B vaccines			
		· Provision of vaccines e.g. rabies and Hep B		

Overall the workshop discussions on the scenarios supported the general trend of more benefits for longer-term/higher risk studies. There was a clear overall agreement that participants should not be made worse off by their participation in any research. It was felt that if the short and low risk activities illustrated in scenarios 1 and 2 in Box 1 had no costs – direct or hidden – it was appropriate not to offer any benefit or payment beyond an appeal to aspirational benefits, and showing common courtesy through for example providing refreshments and research findings, where appropriate. An area of debate (discussed in more detail below) was how much time needs compensation, and what kind of compensation is appropriate for that time. The recommended range of collateral benefits for scenarios 3 and 5, both of which require repeated interactions with well children, were relatively high, to compensate for the relatively large amount of time involved in these studies, and to minimize drop-outs and monitor health. Beyond the payment of direct costs and showing common courtesy, the main benefits considered to be appropriate were diagnosing, treating and referring study children, and providing community benefits such as strengthened dispensary services. The debate (also discussed in greater detail below) was on whether siblings and parents can access health care benefits, and how this is balanced by strengthening community benefits. In scenario 4, there were considered to be significant direct benefits built into the trial design, and significant community benefits as part of wider support to the hospital, and some concern that continuing to increase the collateral benefits to participants might compromise voluntariness in informed consent.

### Experiences with regards to specific benefits, payment and other items

#### Medical benefits

There was a general perception that medical services are often the most appropriate form of benefit given that health care is close to our area of interest and expertise, and that this form of benefit minimizes the move towards a commercialization of the research encounter. All of the seven studies offering benefits provided some form of *direct medical benefit to study participants* (Table 3), with details of the package depending on the study. In addition to free treatment of the disease or problem of interest (for example clinical shock, TB, malaria, RSV, or HIV), study participants usually received free consultation and treatment for other minor or acute illnesses over the course of the study, including out of hours, and in some cases (e.g. PS) without queueing? Other treatment included in most studies were referral of more complex or chronic illnesses identified over the course of the study to other government facilities, including vehicles or transport to the referral facility (PS), costs of doing diagnostic tests e.g. X-rays (HIV) and medical fees (Immuno, MV).

All studies included *collateral medical benefits to non-participants* through for example strengthening of the general services of a facility for all patients with a similar problem. Specific support offered included improved laboratory facilities for TB tests (TB); provision of emergency care training for all intensive care ward medical staff (PS), provision of weighing scales, haemacue machines, i-STAT, and thermometers (PS); and provision of medical staff and refurbishment of existing health facilities (MV).

The audit and workshop group discussions highlighted several challenges related to medical benefits in studies. One set of concerns was *how much of a benefit individuals actually get from a specific study beyond standard of care*. The first challenge is that the standard of care in the paediatric wards is already high relative to other district hospitals as a result of programme wide support (see background); support that is described by some senior researchers both as a collateral benefit and as a necessity for researchers to feel comfortable to conduct research in an area with so many unmet health needs, and to minimise inducement. The overlaps between standards of care, collateral benefits and study requirements were illustrated in an interview:

"We took that approach right from the beginning that we would just first set up a platform for TB diagnosis in Kilifi hospital and which is fundamental to doing the study, but also pretty fundamental to the care for children so we set up this platform and we have made it part of standard of care for children with […] so any child who is referred with suspected TB or is admitted with symptoms and signs consistent with suspected TB gets worked up for TB (IDI No. 1, Researcher)."

Despite the relatively high standard of care, sometimes contributed to by individual studies, it was argued that studies often do involve greater observation and investigation in study children, and that this can lead to improved diagnosis and care even within a relatively well equipped ward.

The second challenge is that while researchers describe free treatment as a benefit, there are national policies for exemption of charges for all children aged less than five years. Nevertheless, it was recognised that across the country there is relatively little adherence to exemption policies [30,31], and therefore that free care remains a significant benefit. When combined with transport, or reimbursed fares (discussed next), the medical benefits were therefore felt to be quite significant especially for the scenarios 3, 4 and 5. PIs working on similar studies reported that non-participants regularly asked to join studies, illustrating that benefits are valued.

Another set of concerns related to medical benefits concerned *who is eligible and where they can seek treatment from*. Regarding the former, the challenge was what to do if parents brought other sick family members for free treatment, or even their neighbour’s children. Interface staff who were approached by individuals not formally eligible for this benefit sometimes found it difficult to refuse a needy case, but were also concerned that providing care would start a precedent for other families and studies. The dilemma could be particularly difficult for members of the index child’s family, with families reportedly commenting that research can introduce unfairness within households, and for example accusations that researchers are interested in the child but not the person who gave birth to him/her:

"In case a parent has been involved in an accident and it is a parent to a study child, the vehicle cannot be offered....they [community members] tell us, “mtoto hakujizaa mwenyewe, alizaliwa na mimi” [this child didn’t give birth to him/herself, I gave birth to him] (FGD no 1; fieldworkers)"

Some study clinicians reported that attending quickly to study children while referring their sick sibling to a long hospital queue felt wrong, and that they would sometimes treat the sibling out of compassion (KI) even where study policy did not support this action. Others would diagnose the non-participants’ problem and then refer them to the hospital pharmacy to buy drugs; thereby at least saving the parent queuing time. But this was difficult where the parent had no money to buy prescribed drugs. Staff described such dilemmas, and having to stick to standard operating procedures (SOPs) set by people who were not present, as incredibly stressful:

"Even we FWs sometimes hurt inside…you may see me growing thin and yet it is stress from the job (laughter)…you are having this enormous burden all on you…(FGD no 1; fieldworkers)"

These challenges were exacerbated by some studies being more inclusive and flexible with regards to assistance of other family members than others. While this would be expected with such different studies, there appeared to be both a lack of standardisation across very similar studies, and problems in communicating clearly about these differences. Related challenges were confusion and sometimes irritation regarding the timing of medical benefits; in some cases participants can receive free treatment at any point over the course of the study (MV), in others it is only when they come back for a specific follow up visit (e.g. PS; TB). The latter can feel short changed, and may be taunted by others:

"…they [non-participants] would tell the participants that, “look, it is only when KEMRI needs to draw blood from your child, that they send you vehicles, but when your child is sick, you trek to hospital just like the rest of us”… (FGD no 1; fieldworkers)"

This phenomenon of local interpretation of perceived differences in benefits between studies leading to confusion and tensions was general to all types of benefits, as discussed more below. One outcome was potential refusal to participate in studies with less tangible benefits:

"[we get told] “No, you are not coming to this house to ask any questions… you take everything [benefits] to the other homes and here you only come to ask questions….No, go to that house… that is your house…(FGD no 2; community facilitators)"

"some would say that, “we are not in the project but we are also seen by the KEMRI doctors (it doesn’t matter that you are seen first)…and we also get lifts-just like you…but only you struggle in the project”… (FGD no 1; fieldworkers)"

At the workshop it was felt that for longer term studies, medical benefits provided should be greater than for short term studies, and that these medical benefits should be extended to participant siblings and other family members wherever possible, not least to help strengthen the relationship with participant families and help ensure retention. There was agreement that overall, support to siblings should at least ensure that the benefits to individuals are not lost through for example a sibling having to queue for care, and that with referrals to other facilities, all costs for the first visit including treatment should be covered. With regards to inter-current illnesses experienced by participants between planned visits in longitudinal studies, for example following discharge from the district hospital, there was a general agreement that these should not be paid for, because there are already significant benefits built into studies, and a requirement for this support would be impossible for many studies.

#### Travel costs

All of the seven studies aimed to ensure that patients did not incur any travel costs (Table 3). Travel costs were therefore either avoided (for example by organising research centre transport to collect people from their homes to facilities; MV study) or refunded. In general, costs were *not* met for travel that would have happened in the absence of the study. For example fares were not refunded for participants in the TB or RSV studies for their first visit to the hospital, because this travel occurred as part of care seeking *before* they were recruited in the study.

There were differences across the studies with regards to how fares were refunded, at what level and what specific costs they were supposed to cover or compensate. For example some studies (e.g. Immuno) would give out money in advance, providing funding at each visit to cover the travel costs of a subsequent attendance. Others (e.g. MV) only refunded return fares on arrival for the research visit. Some studies reimbursed set amounts based on known costs of public transport from the participant’s area of residence, while others refunded actual amounts claimed on the day, in some cases only on production of a receipt. Some studies added a small ‘top up’ to allow for purchase of refreshments for the trip, and some paid a small fixed amount of money to those who walked to the facility because they lived close by or could not access public transport. One study (HIV) incorporated into standard ‘travel costs’ some compensation for time spent on the research activity.

Another difference across studies was exactly who had fares covered, and for what services. For example, costs could include those for the child only, or other siblings that the parent might need to travel with, and for one or two parents; and sometimes studies allowed for transport costs in unexpected health emergencies. Emergency assistance was generally offered only in consultation with health facility nurses, and only by the longer term studies out of normal working hours, primarily as a ‘*humanitarian response*’ (MV) or as a way of ‘*being part of the community*’ (Immuno).

"‘We have been encouraged by the MoH not to set up a parallel system… [they] were very categorical that if somebody is unwell they should follow the normal procedure- go to the nurse in the health facility who will call the ambulance…also because of medico-legal reasons…the ambulance comes with a nurse…there is some sort of first aid at hand…(IDI no 8; researcher)"

The audit revealed significant challenges with (re)payment of travel costs, including concerns associated with different approaches across studies, and between the institution and others operating in the area:

"‘Even with fares; a study will give exact fare, another one will give extra - like one and a half the amount that people are charged, so sometimes it brings problems and you know sometimes they are in one study when they complete then maybe another child is in another study, so they are like, “why is it that I was given double fare and now you are giving me only one way” (laughter)…’ (IDI no 6; clinical officer)"

As for medical benefits, this quote hints at the mistrust that can be introduced by these different approaches, or at least by them being inadequately communicated. Another challenge was introduced by perceived lack of fairness or even sense in not extending transport support beyond the index child in a study, as noted above.

Specific concerns raised with giving fares long in advance of appointments were that:

· where money was spent on other pressing needs, parents would sometimes avoid appointments or otherwise fail to turn up, leading to relatively expensive follow up visits to homes, and possibly embarrassment for families, or

· parents may feel unable to change their minds about coming to follow-up visits, having already accepted the money at an earlier stage.

· Other travel payment concerns were that transport receipts were often difficult for parents to obtain, and that amounts claimed were sometimes higher than those incurred. On the other hand collecting participants from home with research vehicles included the possibility of introducing long waits where arrival time at homes were difficult to predict, and difficulties with handling others needing lifts at the same time. Finally, there were some reports of fieldworkers using their own funds to assist participants and finding it difficult to ask for reimbursement from the study team.

At the workshop it was agreed that transport costs incurred specifically for research must always be paid/reimbursed, and should include a little extra to cover for time spent and other needs on the journey (e.g. drink/snacks). There was a suggestion that to maximise fairness and clarity, to minimise potential to negatively impact on informed consent and to simplify administration systems, studies should:

· Set repayments based on where participants live, regardless of whether they actually use that public transport on that day, and therefore even without receipts. The amounts can be calculated depending on the zone participants live in surrounding research facilities,^a^ with everybody from that zone given the highest expected cost from that zone. Amounts per zone should include a small additional amount to actual expected transport cost, to cover for refreshments or unexpected costs for the journey.

· Allow participants to choose whether they wish to be paid in advance for a future visit, or during that future visit

· Ensure travel is reimbursed for one parent and the index child if he or she is five years or above.

#### Other non-medical benefits

The most common other items offered by studies were food and snacks (five studies) offered when participants were visiting facilities away from home, or – less commonly – when significant amounts of time were taken within households. These were either to compensate for missed meals, as a token of appreciation, or to calm children (sweets and biscuits). Food was provided ready to eat (MV, Immuno, RSV, MV), as money for families to buy their own food (PS), as tickets to exchange for food at selected outlets (HIV), or as dry food for participants to cook themselves as convenient (reported in cross-cutting interviews). Other benefits included notebooks and pens (Immuno) for school-going children. In HIV studies participants were also given T-shirts, lubricants and legal services of a lawyer where charged with minor non-criminal offences such as loitering. Some people also considered the hiring of field workers from the local community as a significant community benefit.

Specific challenges with regards to these benefits (beyond those of other benefits of the amounts and who received these) were reports that some participants felt that very small benefits, juices and biscuits for instance, belittled their contribution, and led to them being taunted by others in the community. A particular concern with regards to t-shirts distributed by one study (HIV) was that some participants reportedly did not want to wear them as they were concerned that they would be stigmatised as having HIV/AIDS.

#### Financial compensation for time

At present, compensation for time is through the inflated fares described (or fares being given where they are not strictly needed), or through medical and other benefits. There were concerns raised through the audit that compensating for time through inflated transport costs, particularly where no transport costs were actually incurred, raised confusion and suspicion. For the HIV studies, there were reports that this approach risks people falsifying information in order to obtain cash, or concerns about confidentiality.

"some would even lie… they would ask, “how come you are there, what did you say?…Okay, these are the kind of people they want?…Okay, I will just go and say this is what I am…I would say I am MSM… I am a sex worker- that is what they want”. (IDI no 7; research manager)."

"Actually for us, maybe one of the things is the variation of reimbursement…you will say that the uninfected cohort, you will give 350… then you have another one being given 500… then you have someone on the trial getting 600… volunteers challenge us…they really want to know why is it that so and so gets 500…and I can’t tell the person what it is about.. I can’t break confidentiality about their status (IDI no 7; research manager)."

While the latter concern may be particularly applicable to research in stigmatised diseases or populations, lack of clarity about what the payments are for, particularly where somebody has walked to a facility, was reported for several studies, and in the workshop. This lack of clarity, in some cases linked to a broader misinformation about what the research is about and how it differs from standard health checks or treatment, can contribute to disputes within households (for example, concerns from husbands about where the mothers of participant children have received money from and why), and fuel rumours around the aims and objectives of the activities (for example, concerns about whether KEMRI is ‘buying blood’).

"…and what that does to the home dynamics… the decision-making…when you give the woman the fare, and ideally a married woman here is only supposed to be supported by the husband…she is only supposed to get money from the husband; so here is KEMRI coming to give her money…exactly what are you doing to the power relations between the woman and the husband…on top of that you are giving her more than she needs for fare…(IDI no 9; community facilitator)"

"[We are told that] “You know very well that there are no matatus [public transport vehicles] in this area yet you say that this is fare…no… just tell us what this money is, but don’t you say that it is fare”…(IDI no 11; nurse)"

It was agreed at the workshop that time costs – including what somebody might be doing in that time, such as preparing a meal or earning an income - should be properly considered in research planning because these costs are often under-estimated. It was recognised for example that families may often stay at the dispensary waiting for a one hour assessment for far longer than an hour, and that field staff may turn up late to a household for an appointment, causing delays. It was agreed that appreciation and common courtesy should be maximised by minimizing inconvenience to participants, and – especially where the participant leaves home - considering the need to provide snacks or lunches. However the question of if and how to provide a reasonable allowance in cash for ‘lengthy’ research activities (either in homes or away from them) was highly complex, given the different types of income generation people have, diverse incomes and the different approaches research staff took to the issues. Given that there was no simple calculation that we could draw on at the workshop, and that there were concerns and debates about moving towards a more commercial relationship with participants/communities, the area was recommended for further careful research and discussion. Subsequently, the small group formed to take forwards workshop findings have suggested that where significant individual time is taken (for example the exceptional cases of an overnight stay in a facility that would not otherwise be necessary), past research in our area supports payment in accordance with national guidelines on minimum daily unskilled wages. At the time of writing this was 300/= or GBP 2.30 per day in an urban setting.

## Discussion

There is general consensus that fair benefits are essential in international health research, but the exact nature of benefits that should be provided to participants and communities, and their mode of provision, are not clearly defined, and often strongly contested. There has been little detailed research on experiences and challenges with benefits on the ground, particularly from low-income settings, to feed into on-going debates. We explored benefits and payments offered for a diverse range of studies within a large long-term multi-disciplinary research programme in Kenya, using both a descriptive and consultative approach. Although not all relevant parties were involved (most notably absent were study participants or their parents, community representatives, health workers and ethics committee members), we were able to include a broad range of programme staff, including the voices of those who are often excluded from policy discussions: the interface staff who explain and administer benefits and payments to research communities; many of whom are from those communities themselves.

We did not cover some types of collateral benefits from studies to communities, such as employment of local personnel, capacity strengthening of researchers from the region, nor post trial benefits. Also not discussed in detail were the collateral benefits such as clinical staff and services funded by the programme to the paediatric wards at the district hospital, and to government health centres and dispensaries in which research is conducted. Nevertheless, we believe that we have gathered sufficient information to begin to draft institutional guidelines on what ‘ought’ to be done in our setting for studies (both procedural and substantive elements), and to highlight some areas that need further research and discussion.

### Challenges with current practice

Our interviews and interactions helped us learn about some of the realities of administering a wide range of benefits and payments on the ground (medical, transport, food and other). A picture emerges of current practice being appreciated by staff and communities, but also being associated with significant issues and challenges which have received little attention in the literature, relative to concerns about undue inducement. Costs to participants that may not be considered for example are lack of common courtesy in research encounters, and amounts of time spent waiting or travelling for research appointments. These costs can mean that participants, rather than being induced into research, are potentially under compensated for their role in research. Concerns of researchers to minimize this possibility and even to introduce a benefit rather than simply a compensation to participants, can contribute to the payment of inflated or unnecessary fares described in our study. The complexity involved in apparently simple reimbursements has recently been noted for another Kenyan setting [32]:

"Underneath the seeming obviousness of the concept of ‘reimbursement’ many other considerations were at play and informally negotiated. These included questions of justice and ethics, and personal commitment to provide some help for poor study subjects, but also budgetary constraints, competition with other groups for participants, and concerns with recruitment rates and participant retention [32; page 50]’."

The transport issues noted above illustrate the complexity in practice in defining benefits, and specifically in distinguishing between benefits and compensation. The transport fare issues in our study also illustrate how benefits and payments can in themselves also exacerbate or introduce new inequities, conflicts and rumours within households and communities, especially where benefits are both significant and visible. Even where benefits have been carefully considered for the participant him/herself in one study, these considerations might be undermined by a household situation (for example if a mother has to queue twice: once for a study participant and again for another sibling), or by other studies being conducted by the same organisation (for example another similar study offering different levels and types of benefits). In this way, the wider research and social context is key to the way that study specific benefits work out in practice. For researchers, these realities may be both obscure and complex, supporting the need for locally relevant institutional guidelines aimed at ensuring that participants are adequately compensated, that intended benefits actually reach participants and communities, and that wider concepts of fairness are supported.

### A middle ground between micro and macro justice issues in approaches to benefits

It became clear over the course of the audit and workshop that research staff generally approach benefits and payments deliberations in two ways: a) focusing on *ensuring that participants do not incur overall costs,* with their overall approach being one of research being based on good will and partnership between researchers and research participants or communities, underpinned by a concern about moving away from this type of relationship towards a more commercial one; or b) aiming to *maximise benefits as far as possible to participants*, given the relative wealth of the institution and poverty of many community members/research participants.

Overall, an appropriate compromise or middle ground that emerged for our context was ensuring that direct benefits to individuals are primarily medical (rather than financial or for example food), and that there is an effort at all times to maximise collateral medical benefits to whole communities, through developing strong collaborations with the Ministry of Health. The collateral benefits can be offered both by specific studies as illustrated by many of our case studies in this paper, but also at a programme wide level through the kind of support that is currently provided as summarised in the institutional section under methods in this paper. This compromise it was hoped would avoid a commercial relationship with participants, and protect and strengthen a key relationship with the MoH. This approach might be considered one way to tackle micro-level justice issues (for example fair benefits and compensations for research participants) in a way that recognises macro-level justice concerns (for example historical grievances and global inequities that perpetuate poverty and ill health), as one of a set of approaches to benefit-sharing at both the micro and macro levels [12,21,23].

There are three important issues to note in proposing this balance. Firstly, while we are describing here appropriate direct and collateral medical *benefits*, we must recognise that all ‘benefits’ that have to be given to participants or communities to ensure scientific validity, prevent study-related harms or address study related injuries, are not benefits at all. Beyond these basic requirements, many other medical ‘benefits’ described as appropriate by our participants might equally or more accurately be framed as *ancillary care responsibilities or even obligations*.

This is particularly given that we are a long term, well resourced research programme (indicating relatively strong relationships and opportunity and capability to provide care), operating in a low income setting facing significant basic health care challenges (indicating urgency of health care needs) [17,19]. Secondly, we recognise the risk that medical benefits increase the potential for therapeutic misconceptions (TMs) arising [29]. Nevertheless we see this TM potential as emphasising the need for careful communication about studies and any included care or benefits rather than as undermining the arguments to provide the benefit or services. Thirdly, we recognise the importance of very careful negotiations and discussions within the institution, and with key Ministry of Health representatives, to ensure that sustainability of additional support is assured, and even that pre-existing services are not undermined in the longer term.

### Towards institutional guidelines to support clarity and transparency in benefits and payments

The need for flexible guidance to support clarity and transparency in benefits and payments was emphasised as crucial by research staff at all levels. It was felt that institutional guidelines could help develop agreed, consistent and rational approaches and explanations on benefits and payments for the main types of study conducted, minimize differences between similar projects, and thereby reduce perceived unfairness. It was also felt that institutional guidelines would potentially reduce staff anxiety associated with conducting research in situations of poverty and hardship. The need for strengthened communication about benefits and payments, including about differences between different types of studies and reasons for these differences, was also highlighted as essential to minimise perceptions of unfairness. The specific issue of benefits and payments is clearly therefore key to include in broader community engagement plans and consent SOPs for studies and the programme overall [28,33].

We are developing guidelines aimed at ensuring fair reimbursement and benefits for research participants and communities. In these guidelines, for all studies researchers will be prompted to:

· Carefully consider the real time, inconvenience and expenses for participants to ensure that these are not underestimated

· Ensure that common courtesy is demonstrated throughout studies by for example minimising inconvenience, ensuring good communication, and providing refreshments.

· Where payments are made, base these on time, inconvenience and expenses for each study and participant, rather than on a flat rate. In calculating fare reimbursements to local residents, offer rates based on zones surrounding a given location, with a small allowance for refreshments, and make payments regardless of production of a receipt. In this approach we differ with that of for example the South African Medical Research Council guidelines which set a flat rate policy per visit for clinical trial participants. The flat rate approach has been variously critiqued for being excessive, inappropriate, unaffordable to non-industry funded trials, and ultimately unfair: participants are paid the same amount but do not do the same things or incur the same expenses [20].

· Recognise that individual participants want their contributions to research to be adequately acknowledged, and that what we might be currently calling medical benefits are in fact responsibilities or obligations in our setting (discussed more below). Ensure that actual benefits for individuals are weighed against potentially introducing intra-community conflicts, and that institutional emphasis IS PLACED where possible ON providing health benefits to broader communities (through discussions with MoH managers and other relevant stakeholders).

To facilitate thinking about specific benefits in relation to a spectrum of study types our guidelines will distinguish between four types of studies 1) clinical trials; 2) sampling only involving no intervention; 3) observational studies involving no sampling or interventions; and 4) interview only studies. For the shortest and least inconvenient studies (for example, a 45 minute interview only study), the key requirements will be communicating effectively about the study, minimising inconvenience and ensuring common courtesy, including returning results where appropriate, rather than offering study benefits. As participants’ inconvenience and time contribution increases, increasing benefits are likely to be appropriate, for example for clinical trials (such as phase 2b and 3 vaccine/drug trials with prolonged follow up) and longer term observational studies. The main argument for increasing benefits for these studies in our setting is linked to concepts of compensation and for a stronger requirement for reciprocity in the studies with longer and more intensive relationships [34]. At the same time, we are aware of a counter-argument that this might introduce a perceived or real unfairness across studies (with those we are more engaged with receiving for example better health care), and that participant ability to withdraw might be compromised [35]. Furthermore, it is not necessarily straightforward to argue that duration and intensity of relationship should influence research participants’ benefits [36]. We try to consider and counter these concerns through strengthening wherever possible programme wide and study specific community benefits, as described above. A relatively complex area that needs further work is how much time needs compensation, and what form of compensation is due for that time. Given the overall concern with setting precedents and distorting the research relationship into a commercial one, this will need particularly careful review and monitoring.

We plan to finalise our guidelines, and check these with research staff and other key stakeholders such as members of the national ERC, community representatives and MoH staff. If agreed, we will begin to implement guidelines with careful monitoring of impacts on individuals, families and communities, and front-line and senior research staff. Importantly, the communication of decisions made regarding benefits and payments will have to be passed within teams to the frontline staff and between research teams and community members as a key part of broader community engagement plans and activities. We hope that this process will help us to continue to learn about this critical, complex and contested area. In so doing we hope we can continue to learn from and feed into experiences and guidelines from other settings.

## Conclusion

We drew on interviews and a consultative workshop to develop draft guidelines for our programme that focus on the direct and collateral benefits offered to study participants and to other community members over the course of the conduct of studies. The difference in benefits and payments currently offered for similar studies in our programme, and some perverse outcomes associated with levels and types of benefits, and how they are administered, support the importance of this initiative for our programme and potentially for other similar settings. Also supporting this initiative is the complexity in distinguishing on the ground between compensation and benefits, and between medical benefits and obligations and responsibilities. We reiterate that consideration of these forms of benefits are only one component of wider considerations at both the micro and macro level of benefit-sharing by individual studies and the institution, including post-study benefits, aspirational benefits from research, and capacity strengthening in research and of health systems.

## Endnotes

^a^Zones to be created for areas with similar known costs to the facility

## Nodes

^1^ Zones to be created for areas with similar known costs to the facility

## Competing interests

The authors declare that they have no competing interests.

## Authors’ contributions

S Molyneux and V Marsh were involved in the conception and design of the study. S Molyneux, V Marsh and S Mulupi participated in data collection, analysis and report write up, while L Mbaabu contributed to literature review and conceptual thinking. S Molyneux wrote the first draft of the paper; all authors commented on drafts, and read and approved the final manuscript.

## Acknowledgements and funding

We are grateful to all of the research and hospital staff who participated in the interviews and workshop discussions. Special thanks to Professor Mike Parker, who facilitated the workshop in Kilifi, and to those who assisted with the workshop preparation and post workshop discussions, including Salim Mwalukore (Community Liaison Manager), Francis Kombe (Community Facilitator), Patricia Njuguna (Clinician/researcher), Sam Kinyanjui (Head of Training), and Dorcas Kamuya (Social scientist). This work was supported by the Wellcome Trust, UK (WT085418 to S.M; 089316/Z/09/Z to V.M.; GR074314MA to VM/DK/SM) and the Kenya Medical Research Institute, Kenya. This article is published with the permission of the director, KEMRI.

## Pre-publication history

The pre-publication history for this paper can be accessed here:

http://www.biomedcentral.com/1472-6939/13/13/prepub
